# SopB activates the Akt-YAP pathway to promote *Salmonella* survival within B cells

**DOI:** 10.1080/21505594.2018.1509664

**Published:** 2018-08-26

**Authors:** Abraham García-Gil, Carlos Samuel Galán-Enríquez, Araceli Pérez-López, Porfirio Nava, Celia Alpuche-Aranda, Vianney Ortiz-Navarrete

**Affiliations:** a Departamento de Biomedicina Molecular, Centro de Investigación y de Estudios Avanzados del Instituto Politécnico Nacional, Ciudad de México, México; b Department of Pediatrics, University of California San Diego, San Diego, CA, USA; c Departamento de Fisiología, Biofísica y Neurociencias, Centro de Investigación y de Estudios Avanzados del Instituto Politécnico Nacional, Ciudad de México, México; d Centro de Investigación Sobre Enfermedades Infecciosa, Instituto Nacional de Salud Pública, SSA, Cuernavaca, México

**Keywords:** *Salmonella*, B cells, SopB, Akt, YAP, IL-1β

## Abstract

B cells are a target of *Salmonella* infection, allowing bacteria survival without inducing pyroptosis. This event is due to downregulation of *Nlrc4* expression and lack of inflammasome complex activation, which impairs the secretion of IL-1β. YAP phosphorylation is required for downregulation of *Nlrc4* in B cells during *Salmonella* infection; however, the microorganism’s mechanisms underlying the inhibition of the NLRC4 inflammasome in B cells are not fully understood. Our findings demonstrate that the *Salmonella* effector SopB triggers a signaling cascade involving PI3K, PDK1 and mTORC2 that activates Akt with consequent phosphorylation of YAP. When we deleted *sopB* in *Salmonella*, infected B cells that lack Rictor, or inhibited the signaling cascade using a pharmacological approach, we were able to restore the function of the NLRC4 inflammasome in B cells and the ability to control the infection. Furthermore, B cells from infected mice exhibited activation of Akt and YAP phosphorylation, suggesting that *Salmonella* also triggers this pathway *in vivo*. In summary, our data demonstrate that the *Salmonella* effector inositide phosphate phosphatase SopB triggers the PI3K-Akt-YAP pathway to inhibit the NLRC4 inflammasome in B cells. This study provides further evidence that *Salmonella* triggers cellular mechanisms in B lymphocytes to manipulate the host environment by turning it into a survival niche to establish a successful infection.

## Introduction

*Salmonella enterica* serovars are gram-negative bacteria that are able to infect a broad range of hosts and cause both acute and chronic infections [1]. It is estimated that *Salmonella enterica* serovar Typhi is responsible for 21.7 million of new infections worldwide annually [2]. Moreover, approximately 2–5% of patients are not able to fully clear the infection and become chronic carriers [3]. In contrast, most non-Typhi *Salmonella* serovars (NTS) cause self-limiting gastroenteritis in immunocompetent humans and are some of the most important microorganisms causing food-borne diseases worldwide [4]. Therefore, *Salmonella* infection remains a public health concern, and studies on the mechanisms involved in these infections remain important.

Macrophages have been considered the main target of *Salmonella* during infection, and these cells are responsible for bacterial dissemination and control [5]. In addition to macrophages, other cells of the immune system are targets of this pathogen, including dendritic cells and neutrophils [6,7]. Furthermore, we and others have reported that B cells are also a target of *Salmonella* [8–12]. Thus, this pathogen is able to infect a wide range of cell types due to a wide range of virulence determinants, such as pathogenicity islands and virulence plasmids [5,13]. So far, 23 *Salmonella* pathogenicity islands (SPIs) have been described [14], with SPI-1 and SPI-2 being highly required for *in vivo* infection. SPI-1 is involved in epithelial cell invasion and it also involved in post-invasion processes, while SPI-2 is necessary for intracellular survival in the host [15]. In addition, both SPI-1 and SPI-2 encode a type-three secretion system (T3SS), which is a molecular machine involved in the translocation of virulence effectors across membranes into host cells [5].

SPI-1 is required for invasion during oral infection, and the effectors encoded in this pathogenicity island are involved in cytoskeleton rearrangements of epithelial cells to promote the entry of *Salmonella* via macropinocytosis [16,17]. SopE/SopE2 and the inositide phosphate phosphatase SopB are some of the effector proteins responsible for the induction of macropinocytosis. SopB is also involved in host cell survival through activation of the Akt signaling pathway [16,18,19]. Additional functions have recently been described for SPI-1, including activation of the host innate immune system and induction of cell death [20,21]. SipA induces the recruitment of polymorphonuclear cells across the epithelial barrier [21], while SipB is involved in the induction of pyroptotic cell death in macrophages [20]. Moreover, the T3SS encoded by SPI-1 (T3SS-1) is required to activate the Nod-like receptor (NLR) family CARD domain-containing protein 4 (NLRC4) inflammasome complex in macrophages [22].

Once *Salmonella* enters the cell, flagellin and some components of the T3SS-1 reach the cytoplasm and interact with NLRC4, leading to NLRC4 inflammasome activation and IL-1β/IL-18 processing and secretion [22,23]. Thus, activation of NLRC4 requires a two-hit process to induce IL-1β secretion: the first hit is the induction of pro-IL-1β and pro-IL-18 synthesis through activation of Toll-like receptor (TLR), and the second hit is the initiation of inflammasome assembly, which initiates caspase-1 self-cleavage and formation of the active heterotetrameric caspase-1. This cysteine-aspartic acid protease activates several proteins, including pro-IL-1β and pro-IL-18, and induces the secretion of both cytokines [24].

In addition to IL-1β/IL-18 secretion, macrophage cell death via pyroptosis is induced by activation of the NLRC4 inflammasome [20]. In contrast, *Salmonella*-infected B cells do not secrete IL-1β/IL-18 and do not undergo pyroptosis. Downregulation of *Nlrc4* transcription and lack of caspase-1 activity in *Salmonella*-infected B cells have been associated with these findings [25]. Transcription of the *Nlrc4* gene is controlled by the p73-YAP heterodimer. Yes-associated protein (YAP) is a pro-apoptotic transcriptional coactivator that acts within the Hippo pathway and regulates cell proliferation, cell differentiation, spatial organ patterning and tissue regeneration [26]. Furthermore, YAP potentiates p73 function as a transcription factor [27–29]. Additionally, YAP has been reported as a potential integrator of cell death processes and autophagy during cellular stress [30–32]. When YAP is phosphorylated at serine 127, it remains in the cytoplasm and is unable to interact with p73, resulting in impaired transcription of the *Nlrc4* gene [25,33]. *Salmonella* induces YAP phosphorylation during B cell infection, triggering the transcriptional downregulation of the *Nlrc4* gene [25]. Although the mechanism of NLRC4 inflammasome inhibition in B cells during *Salmonella* infection is partially understood, the bacterial effector(s) and mechanism(s) involved in this event and/or the further consequences of YAP phosphorylation are still unknown.

In this study, we show that the bacterial effector SopB activates Akt and then YAP phosphorylation; as a result, transcription of the NLRC4 inflammasome is inhibited in B cells, and consequently, there is no IL-1β secretion or pyroptosis.

## Results

### *Salmonella* promotes Akt activation in B cells

**Figure 1.**
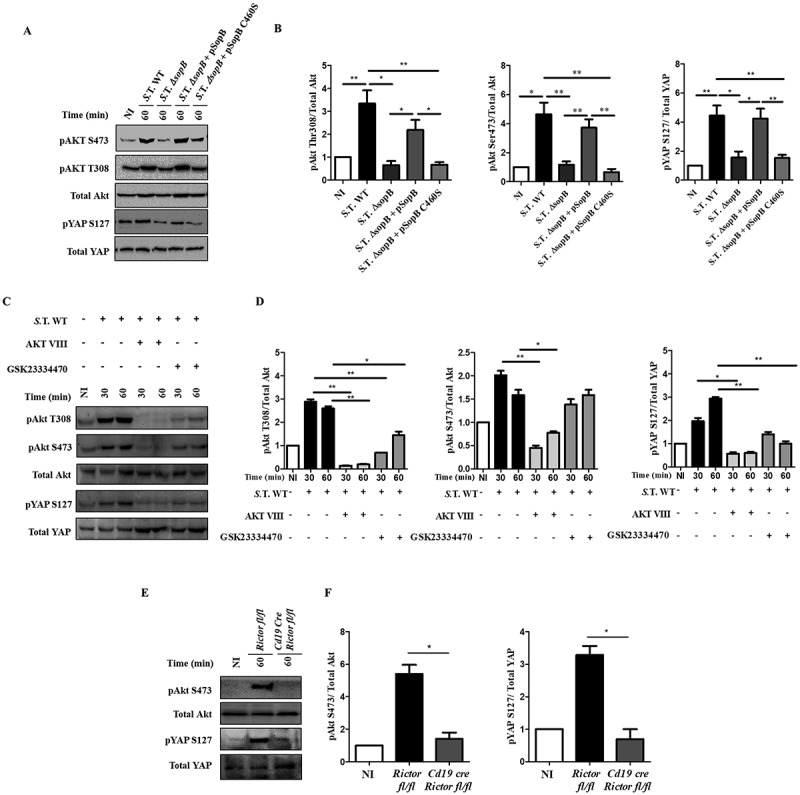
Salmonella effector SopB promotes Akt activation and YAP phosphorylation in B cells. (a) Western blot analysis of pAkt S473, pAkt T308, pYAP S127 and the corresponding total protein levels in B cells from BALB/c mice infected with *Salmonella* Typhimurium wild-type, *Salmonella* Typhimurium *ΔsopB, Salmonella* Typhimurium *ΔsopB-*pSopB or *Salmonella* Typhimurium *ΔsopB-*pSopB C460S at an MOI of 50. (b) Protein levels of pAkt S473, pAkt T308 and pYAP relative to total Akt or total YAP levels were normalized to the control (not infected). (c) Western blot analysis of pAkt S473, pAkt T308, pYAP S127 and the corresponding total protein levels in B cells from BALB/c mice pretreated for 30 minutes with the Akt inhibitor AKT VIII (2,12 µM) or the PDK1 inhibitor GSK23334470 (50 µM) and infected with *Salmonella* Typhimurium wild-type at an MOI of 50. Inhibitors were present before, during and after infection. (d) Protein levels of pAkt S473, pAkt T308 and pYAP S127 relative to total Akt and total YAP levels were normalized to the control (not infected). (e) Western blot analysis of pAkt S473 and pYAP S127 and the corresponding total protein levels in B cells from *Rictor^fl/fl^* and *Cd19Cre*/*Rictor^fl/fl^* mice infected with *Salmonella* Typhimurium wild-type at an MOI of 50. (f) Protein levels of pAkt S473 and pYAP S127 relative to total Akt and total YAP levels were normalized to the control (not infected). Data are representative Western blotting images or are expressed as the mean ± S.D. of at least three different experiments. Data were analyzed by Student’s *t*-test. *p < 0.05, **p < 0.01, ***p < 0.001.

It has been demonstrated that Akt can phosphorylate YAP at serine 127 [34], leading to the translocation of YAP to the cytoplasm from the nucleus, in which it functions as a coactivator of transcription factors, including p73. To identify the signal involved in the downregulation of NLRC4, we hypothesized that the inositide phosphate phosphatase SopB, which is produced by *Salmonella*, may promote YAP phosphorylation through Akt in infected B cells. When we infected B cells with *Salmonella*, Akt was fully activated at threonine 308 (pAkt-T308) and serine 473 (pAkt-S473) (Figure 1(a,b); Figure S1(a,c)). Remarkably, we normally observe that most of those infected B cells harbour single bacteria (Figure S1(a,b)). Importantly, Akt activation was not only observed in B cells that internalized bacteria but also in cells that did not internalize bacteria (Figure S1(d)), suggesting that close contact of *Salmonella* with the surface of B cells was sufficient to induce phosphorylation. To confirm that SopB is involved in Akt activation, we generated a *Salmonella* Typhimurium Δ*sopB* mutant. This mutant strain has similar capacity as *Salmonella* wild type to infect B cells since we observed with both strains between 7–8% of infected B-cells. (Figure S1(e)). When B cells were infected with *Salmonella ΔsopB*, we did not observe an increase in Akt activation. In contrast, when the isogenic Δ*sopB* strain was complemented with a plasmid that expresses the *sopBsigE* operon [35] under control of the *sopB* promotor (pSopB), Akt activation was restored in infected B cells. Furthermore, complementation of the *ΔsopB* strain with a plasmid that expresses a catalytically inactive phosphatase mutant of SopB (pSopB C460S) did not restore Akt activation (Figure 1(a,b)). These results establish that SopB phosphatase activity is required for *Salmonella*-mediated Akt phosphorylation in infected B cells (Figure 1(a,b); Figure S1(b,c)).

### YAP phosphorylation is dependent on SopB-Akt

To evaluate whether SopB-dependent Akt activation was responsible for YAP phosphorylation during *Salmonella* infection, we detected the presence of pYAP-S127 in B cells infected with *Salmonella* wild-type or *Salmonella ΔsopB*. YAP phosphorylation was observed in B cells infected with *Salmonella* wild-type (WT) but not in B cells infected with *Salmonella ΔsopB* (Figure 1(a,b). YAP phosphorylation was restored when *Salmonella ΔsopB* was complemented with the pSopB plasmid but not when it was complemented with the pSopB-C460S plasmid (Figure 1(a,b)). Furthermore, the A20 B cell line transfected with a *sopB*-encoding plasmid (pcDNA6-*sopB*) showed strong activation of the Akt-YAP pathway (Figure. S2). These data demonstrate that the phosphatase activity of SopB is required for YAP phosphorylation.

In addition, we infected B cells in the presence of the phosphorylation inhibitors AKT VIII (Akt inhibitor) or GSK23334470 (inhibitor of the phosphoinositide-dependent-kinase 1 (PDK1)) [36,37]. Both inhibitors blocked the phosphorylation of YAP in B cells infected with *Salmonella* WT (Figure 1(c,d). To strengthen the role of Akt in YAP phosphorylation during *Salmonella* infection of B cells, we generated a conditional knockout mouse for the *Rictor* gene, which is part of the mTORC2 complex responsible for phosphorylating Akt at serine 473 [38]. (Figure S3). As expected, neither pAkt-S473 nor pYAP-S127 was detected in *Rictor*^−/-^ B cells infected with *Salmonella* (Figure 1(e,f)); thus, mTORC2 is required to fully activate Akt and YAP phosphorylation. In conclusion, *Salmonella* SopB promotes Akt activation to phosphorylate YAP.

### *Salmonella*-dependent Akt activation in B cells follows a canonical PI3K activation mechanism

**Figure 2.**
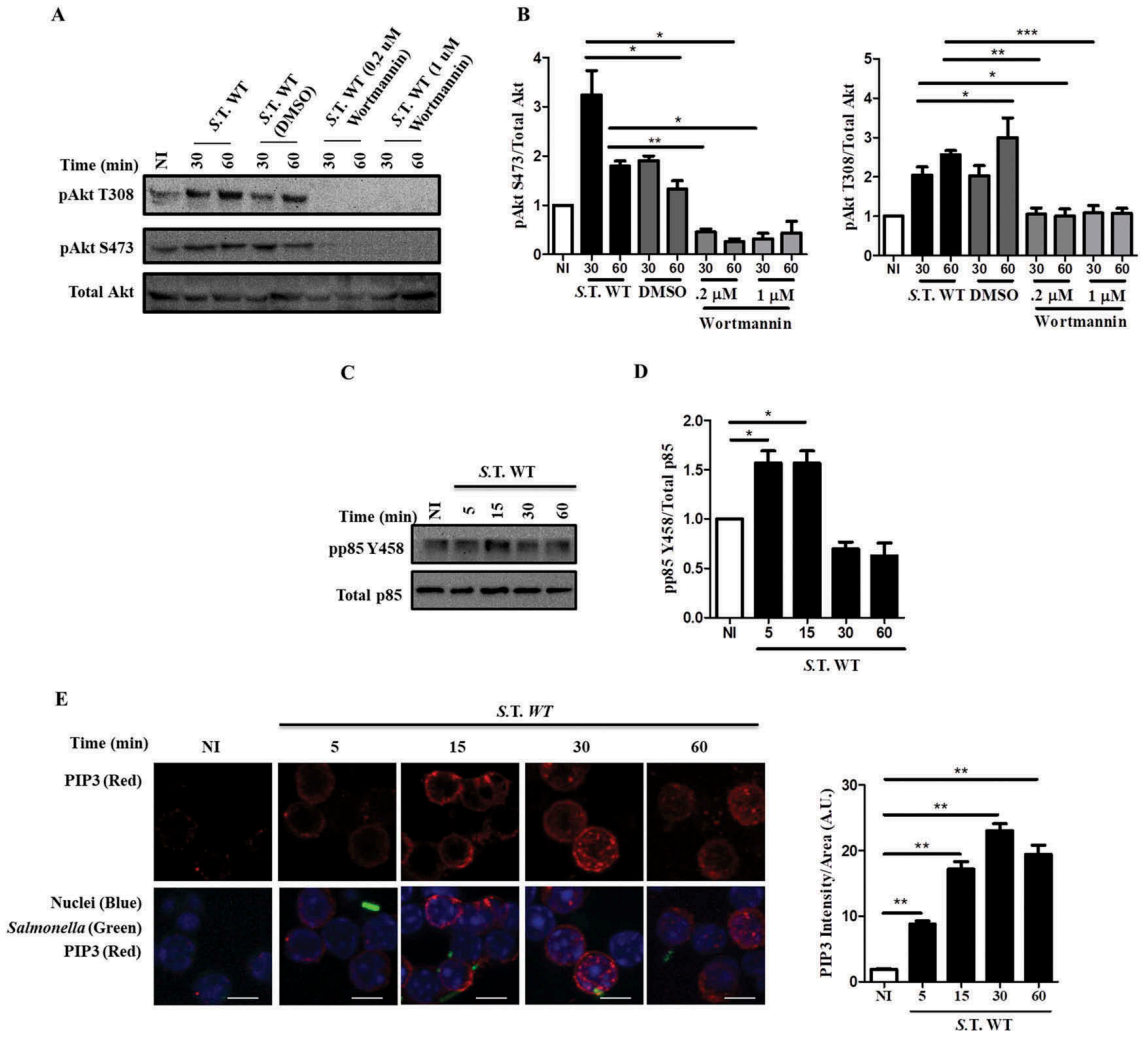
Activation of Akt by *Salmonella* in B cells follows a canonical PI3K activation mechanism. (a) Western blot analysis of pAkt S473, pAkt T308 and total Akt in B cells from BALB/c mice pretreated for 60 minutes with the PI3K inhibitor wortmannin at a concentration of 0,2 µM or 1 µM and infected with *Salmonella* Typhimurium wild-type at an MOI of 50. (b) Protein levels of pAkt T308 and pAkt S473 relative to total Akt levels were normalized to the control (not infected). (c) Western blot analysis of p-p85 Y458 and total p85 in B cells from BALB/c mice infected with *Salmonella* Typhimurium wild-type at an MOI of 50. (d) Protein levels of p-p85 Y458 relative to total p85 levels were normalized to the control (not infected). (e) Representative confocal immunostaining of PIP3 in B cells from BALB/c mice infected with *Salmonella* Typhimurium wild-type-GFP at an MOI of 50. Data are representative Western blotting images or are expressed as the mean ± S.D. of three different experiments. Data were analyzed by Student’s *t*-test. *p < 0.05, **p < 0.01, ***p < 0.001. Scale bar, 5 µm.

Akt activation requires phosphatidylinositol (3,4,5) triphosphate (PIP3) production by phosphoinositide kinase 3 (PI3K). PIP3 production by PI3K leads to Akt recruitment to the plasma membrane and its activation [39]. In HeLa cells, Akt activation by SopB is class I PI3K independent, while it is class I PI3K dependent in mouse embryonic fibroblasts (MEFs) [40,41]. We evaluated if Akt activation by SopB in B cells requires class I PI3K. When B cells were infected with *Salmonella* WT in the presence of the PI3K inhibitor wortmannin, Akt was not phosphorylated (Figure 2(a,b)). PIP3 production was measured by phosphorylation analysis of the regulatory subunit of class IA PI3K p85. We found that p85 was phosphorylated during early infection, correlating with robust PIP3 production in B cells infected with *Salmonella* wild-type (Figure 2(c,d)). In parallel, these infected B lymphocytes were analyzed using immunofluorescence and confocal microscopy to detect PIP3 production, and the results demonstrated that infection increased PIP3 accumulation in *Salmonella*-infected cells (Figure 2(e)). Therefore, Akt activation in *Salmonella*-infected B cells follows the well-recognized PI3K activation pathway, followed by PIP3 production.

### *Salmonella* promotes Akt-YAP pathway activation to block IL-1β secretion

**Figure 3.**
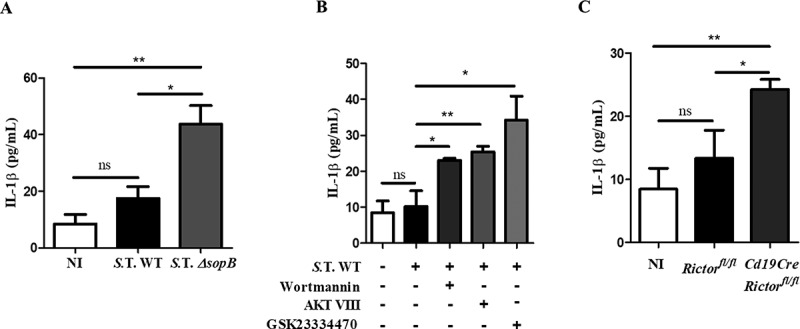
Salmonella promotes Akt-YAP pathway activation to block IL-1β secretion. (a) IL-1β secretion levels in supernatants of B cells from BALB/c mice infected with *Salmonella* Typhimurium wild-type or *ΔsopB* at an MOI of 50. (b) IL-1β secretion levels in supernatants of B cells from BALB/c mice pretreated for 60 minutes with the PI3K inhibitor wortmannin (0.2 µM) or for 30 minutes with the Akt inhibitor AKT VIII (2,12 µM) or the PDK1 inhibitor GSK23334470 (50 µM) and infected with *Salmonella* Typhimurium wild-type at an MOI of 50. Inhibitors were present before, during and after infection. Viability of infected-B cell not treated with inhibitors was similar to infected-B cell treated with inhibitors (data not shown) (c) IL-1β secretion levels in supernatants of B cells from *Rictor^fl/fl^* and *Cd19Cre*/*Rictor^fl/fl^* mice infected with *Salmonella* Typhimurium wild-type at an MOI of 50. Data are expressed as the mean ± S.D. of three different experiments. Data were analyzed by Student’s *t*-test. *p < 0.05, **p < 0.01, ***p < 0.001.

Once we had determined that SopB-Akt are responsible for YAP phosphorylation, we evaluated if SopB is required to block IL-1β secretion in B cells. We found that in contrast to B cells infected with *Salmonella* WT, those infected with *Salmonella ΔsopB* showed restoration of IL-1β secretion (Figure 3(a)). This result confirms that SopB is necessary to block IL-1β secretion in B cells. Quantification of IL-1β secretion in *Salmonella*-infected B cell supernatants from *Rictor*^−/-^ or wild-type mice in the presence of the inhibitors wortmannin, AKT VIII, or GSK23334470 demonstrated that Akt partially (GSK23334470, *Rictor*^−/-^) or fully blocked (wortmannin, AKT VIII) activation. Additionally, the secretion of IL-1β was restored to levels comparable to those in B cells infected with *Salmonella* Δ*sopB* (Figure 3(b,c)). These results support the hypothesis that SopB is the *Salmonella* effector responsible for activation of the PI3K-Akt-YAP pathway and regulation of IL-1β production in B cells. In addition, SopB induced IL-10 secretion and blocked IL-6 production (Figure S4(a,b)), which together with the blockade of IL-1β production would promote an anti-inflammatory environment.

### *Salmonella* activates the Akt-YAP pathway through SopB to survive in B cells

**Figure 4.**
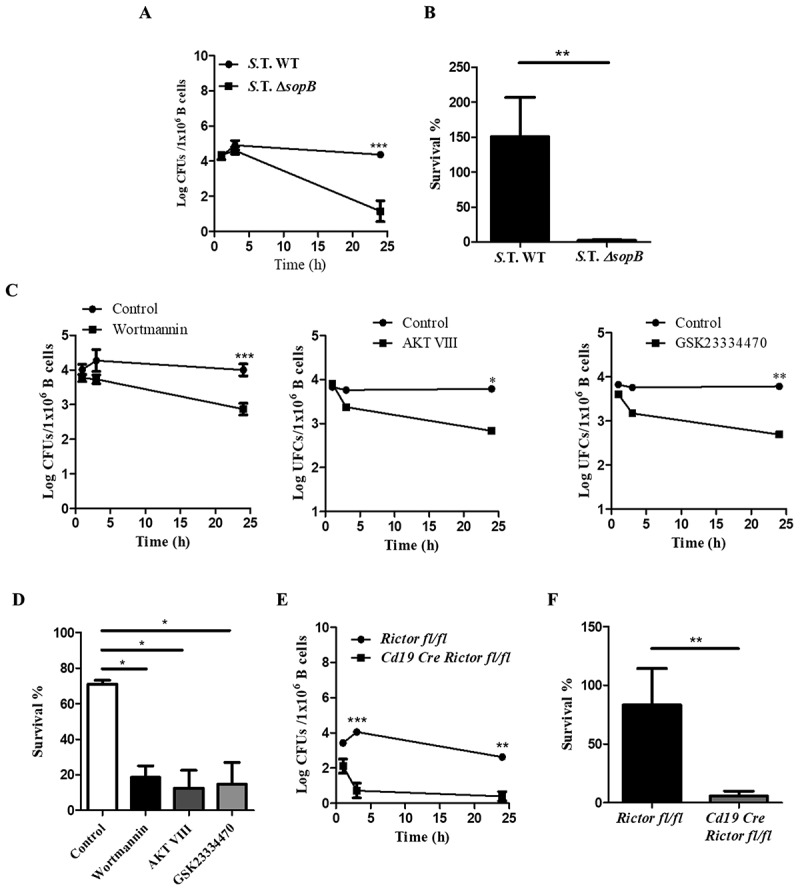
Salmonella activates the Akt-YAP pathway through SopB to survive in B cells. (a) Bacteria kinetic survival in B cells from BALB/c mice infected with *Salmonella* Typhimurium wild-type or *Salmonella* Typhimurium *ΔsopB* at an MOI of 50. (b) Survival percentage was calculated using the following formula (CFUs 24 h PI/CFUs 1 h PI). (c) Bacteria kinetic survival in B cells from BALB/c mice pretreated for 60 minutes with the PI3K inhibitor wortmannin (0.2 µM) or for 30 minutes with the Akt inhibitor AKT VIII (2,12 µM) or the PDK1 inhibitor GSK23334470 (50 µM) and infected with *Salmonella* Typhimurium wild-type at an MOI of 50. Inhibitors were present before, during and after infection. Viability of infected-B cell not treated with inhibitors was similar to infected-B cell treated with inhibitors (data not shown) (d) Survival percentage was calculated using the following formula: (CFUs 24 h PI/CFUs 1 h PI). (e) Bacteria kinetic survival in B cells from *Rictor^fl/fl^* and *Cd19Cre*/*Rictor^fl/fl^* mice infected with *Salmonella* Typhimurium wild-type at an MOI of 50. (F) Survival percentage was calculated using the following formula: (CFUs 24 h PI/CFUs 1 h PI). Data are expressed as the mean ± S.D. of at least three different experiments. Survival percentage data were analyzed by Student’s *t*-test. Survival kinetic data were analyzed by two-way ANOVA with a post hoc Bonferroni test. *p < 0.05, **p < 0.01, ***p < 0.001.

To determine whether SopB is required for *Salmonella* survival within B cells, we infected B lymphocytes with *Salmonella* WT or *Salmonella* D*sopB* and then evaluated the intracellular survival of bacteria at different times. Both the *Salmonella* WT and D*sopB* strains showed similar kinetics at 1 and 3 h post-infection; however, *Salmonella* D*sopB* exhibited a 3.2-log decrease in CFUs within B cells at 24 hours post-infection (Figure 4(a,b); Figure S5). *Salmonella* WT survival within B cells pretreated with a PI3K, PDK1 or Akt inhibitor decreased similarly to that in B cells infected with the *ΔsopB* strain (1.5 logs, 1.6 logs, and 1.5 logs, respectively) (Figure 4(c,d)). In *Rictor*^−/-^ B cells, *Salmonella* survival was strikingly reduced, exhibiting a very low survival percentage (only 0.39 logs of CFU recovered) (Figure 4(e,f)). These results demonstrate that *Salmonella* SopB, through PI3K, PDK1 and mTORC2, activates the Akt-YAP pathway, and this mechanism allows its survival within B cells.

### SopB promotes Akt-YAP pathway activation *in vivo*

**Figure 5.**
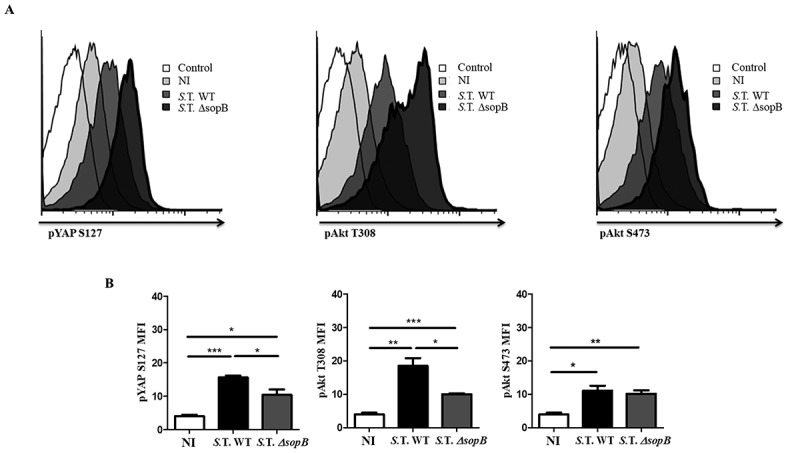
Salmonella SopB activates the Akt-YAP pathway in B cells infected in vivo. (A) Flow cytometric analysis of pAkt S473, pAkt T308 and pYAP S127 in B cells from BALB/c mice infected intraperitoneally with 50 CFUs of *Salmonella* Typhimurium wild-type-GFP or *Salmonella* Typhimurium *∆sopB-*GFP. (B) Mean fluorescence intensity of pAkt S473, pAkt T308 and pYAP S127. Data are expressed as the mean ± S.D. of three different experiments. Data were analyzed by Student’s *t*-test. *p < 0.05, **p < 0.01, ***p < 0.001.

BALB/c mice were intraperitoneally infected with fifty bacteria, either *Salmonella* WT-GFP or *Salmonella* Δ*sopB-*GFP, as established in our previous work [42,43]. Eighteen hours post-infection, we evaluated Akt activation and YAP phosphorylation in splenic B cells using flow cytometry. We found 50% higher YAP phosphorylation and 78% higher Akt phosphorylation at T308 in B cells from mice infected with *Salmonella* WT than in those from mice infected with *Salmonella ΔsopB* (Figure 5(a) left and center panels, 5B left and center panels). However, we observed only 8% higher Akt phosphorylation at S473 (Figure 5(a) right panel, 5B right panel). In addition, Akt-YAP pathway activation was observed in only infected B cells (GFP^+^) (Figure S7), corroborating the notion that SopB is responsible for efficiently activating the Akt-YAP pathway in *Salmonella*-infected B cells *in vitro* and *in vivo*.

**Figure 6.**
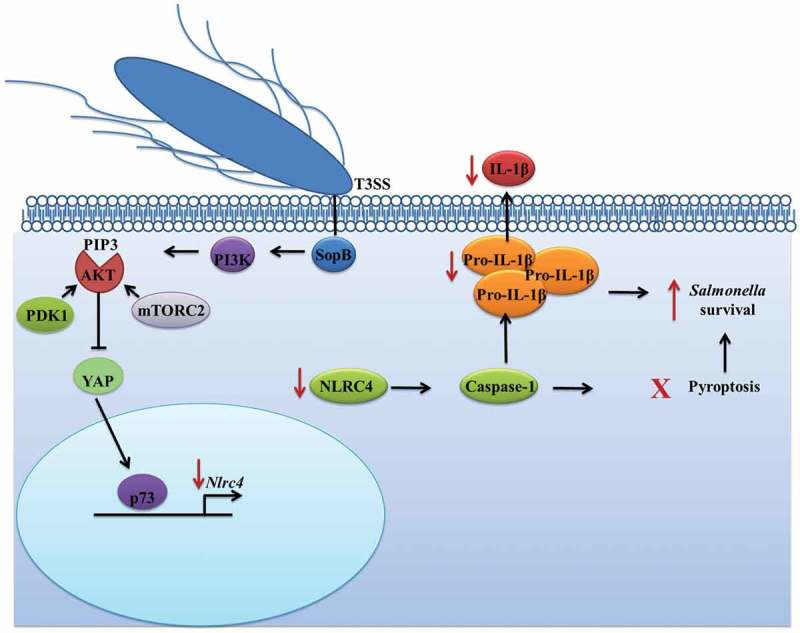
Salmonella effector SopB activates the Akt-YAP pathway to prevent IL-1β secretion. Through PI3K, SopB promotes the production of PIP3, which recruits Akt. Next, PDK1 and mTORC2 phosphorylate Akt (at threonine 308 and serine 473, respectively) to activate it. Once active, Akt phosphorylates the coactivator YAP at serine 127, leading to YAP sequestration in the cytoplasm and preventing it from heterodimerizing with the transcription factor p73. Without the p73-YAP heterodimer, the transcription of *Nlrc4* is reduced, which impacts the processing and secretion of the pro-inflammatory cytokine IL-1β and leads to the survival of *Salmonella* in B cells.

## Discussion

SopB is an inositol phosphate phosphatase that plays an important role in cell invasion and enteropathogenesis and is involved in protecting epithelial cells from apoptosis through Akt activation [16–19,44]. Likewise, SopB promotes the accumulation of PI(3,4)P2/PI(3,4,5)P3 at the cell membrane, which leads to Akt recruitment and activation by PDK1 and mTORC2. In this study, we showed that during *Salmonella* infection of primary B cells, SopB mediates the membrane accumulation of PIP3 in a process that requires class I PI3K. Moreover, Akt is also activated by PDK1 and mTORC2. Our results demonstrated an additional role of fully activated Akt in phosphorylating the transcriptional coactivator YAP. When YAP is phosphorylated, it is retained in the cytoplasm and is unable to induce the transcription of *Nlrc4*, with a consequent reduction in IL-1β production (Figure 6). It was recently reported that SopB inhibits NLRC4 inflammasome activation in bone marrow-derived macrophages (BMDMs), specifically preventing ASC oligomerization, in a process dependent on Akt signaling [45]. Consequently, SopB may trigger more than one signaling pathway to inhibit NLRC4 inflammasome activation.

Up to half of host phosphorylation events during *Salmonella* infection have been directly linked to SopB, and this modulation of processes via phosphorylation is key to promote host cell and *Salmonella* survival [46–48]. Our results fully demonstrated that in B cells, *Salmonella* SopB promotes YAP phosphorylation through Akt and plays a role in this signal transduction pathway that allows inhibition of the NLRC4 inflammasome. Considering that YAP is a coactivator that regulates important biological processes such as apoptosis, autophagy and microRNA synthesis [31,32,49], *Salmonella* induction of this event may affect other biological processes in B cells that were not analyzed in this study.

The inhibition of IL-1β production in B cells by *Salmonella* was reported by Perez-Lopez [25]. In this study, we report that the bacterial effector SopB is responsible for this event. Both processes, IL-1β secretion and pyroptosis, are linked, as they are both downstream of caspase-1 activation [23]. In B cells infected with *Salmonella* Typhimurium *ΔsopB*, we observed re-establishment of IL-Iβ secretion; surprisingly, these cells did not undergo pyroptosis (Figure S8). A similar event has been observed in neutrophils and primary human monocytes infected with *Salmonella*; these cells secreted IL-1β without undergoing pyroptosis [6,50]. These results support the idea that IL-1β secretion might be a pyroptosis-independent mechanism. In this context, gasdermin D has recently emerged as an important player for the induction and execution of pyroptosis. This autoregulated protein is responsible for pyroptosis and is active only when processed by caspase-1 or caspase-11 [51,52]. However, recent reports have described gasdermin D processing and the formation of membrane pores to allow IL-1β secretion without inducing pyroptosis [53,54]. The decision between killing the cell or allowing IL-1β secretion might depend on the strength of the signal that promotes inflammasome activation and gasdermin D processing. According to Evavold’s study, pyroptosis and IL-1β release can be uncoupled under “cell hyperactivation”[53]; therefore, we can conclude that B cells infected with *Salmonella ΔsopB* achieve a state of hyperactivity.

In addition to inhibiting IL-1β production, SopB is shown in this study to be involved in limiting IL-6 production and promoting IL-10 production. IL-10 is an anti-inflammatory cytokine that reduces the antimicrobial capacity of macrophages and regulates MHC-II antigen presentation [55]. Thus, *Salmonella* also promotes an anti-inflammatory environment suitable for bacterial survival. Moreover, it has been reported that IL-10 signaling through IL-10R intersects with and amplifies the PI3K-Akt-mTORC1 pathway [56]. mTORC1 is a protein complex that is involved in metabolism and autophagy regulation, and the latter is an important process that enables intracellular bacterial control; therefore, through Akt activation and IL-10 production, *Salmonella* might regulate autophagy to avoid control by the host. It has been reported that B cells produce IL-10 in response to *Salmonella* infection [57]. Thus, it is likely that through autocrine mechanisms, IL-10 also contributes to bacterial survival by blocking xenophagy. Additionally, it has been demonstrated that SopB recruits and activates TRAF6, an ubiquitin ligase responsible for STAT3 ubiquitination [58], allowing phosphorylation and activation. STAT3 is a transcription factor necessary for IL-10 production in macrophages; thus, it is possible that SopB-TRAF6 participates in IL-10 gene transcription in *Salmonella*-infected B cells. In this way, there is an amplifying process that contributes to avoiding bacterial control.

In summary, we demonstrated in this study that SopB leads to activation of the PI3K-Akt-YAP pathway in primary, *Salmonella*-infected B cells. This event regulates cytokine production to promote an anti-inflammatory environment, which supports the hypothesis that *Salmonella* activates pathways that create a friendly intracellular niche for *Salmonella* replication and survival within B cells.

## Materials and methods

### Mouse strains

BALB/c and C57Bl/6 mice were bred at the Unit for Animal Production of the Center for Research and Advance Studies (CINVESTAV), México City, México. Strain STOCK Cd19tm1(cre)Cgn Rictortm1.1Klg was generated by backcrossing the strains STOCK Rictortm1.1Klg/SjmJ (Jackson Laboratories, stock number 020649) and B6.129P2(C)-Cd19tm1(cre)Cgn/J (Jackson Laboratories, stock number 006785). All the mice were used according to institutional guidelines, and the CINVESTAV animal care and use committee approved the animal protocol.

### Primary mouse cells

Splenic B cells were obtained by CD43 negative selection (Miltenyi Biotec, 130-090-862) and were resuspended in RPMI 1640 medium (Life Technologies, 11875-093) supplemented with 10% fetal bovine serum (FBS) (RP10) (HyClone, SH30071.01). For PI3K inhibition assays, B cells where treated with wortmannin (0.2 µM or 1.0 µM) (Sigma W1628) 1 hour before and during the infection. For Akt and PDK1 inhibition assays, B cells were treated with AKT VIII (2.12 µM) (Calbiochem 124018) or GSK23334470 (50 µM) (Sigma SML0217) 30 minutes before and during infection.

### Bacterial strains, growth conditions, and cell culture

*Salmonella enterica* serovar Typhimurium 14,028 (American Type Culture Collection) WT was used in this study. The *Salmonella enterica* serovar Typhimurium Δ*sopB* strain was generated using the lambda red system as described elsewhere (the lambda red system was kindly provided by William Navarre’s laboratory at Toronto University) [59]. In reconstitution experiments, *Salmonella* Typhimurium Δ*sopB* was complemented with a plasmid that expresses the *sopBsigE* operon under control of the *sopB* promotor (pSopB) or with a plasmid that expresses a catalytically inactive phosphatase mutant of SopB (pSopB C460S) (both plasmids were kindly provided by Olivia Steele-Mortimer’s laboratory at the National Institutes of Health). *Salmonella* Typhimurium WT and *Salmonella* Typhimurium *ΔsopB* strains carrying the plasmid pEM180, which expresses GFP under the ampicillin promoter, were grown in the presence of 100 µg/ml ampicillin and used as GFP-expressing *S*. Typhimurium strains (*S*. Typhimurium-WT-GFP, *S*. Typhimurium-*ΔsopB*-GFP). All strains were grown overnight in Luria Bertani (LB) medium (Sigma L1900) at 37ºC with shaking, diluted 1:30 with fresh LB medium, and cultured until the logarithmic phase was reached. Bacterial cells were adjusted to an OD of 0.6 at 600 nm to obtain the desired multiplicity of infection (MOI). All strains were diluted in RP10 medium before *in vitro* infection.

### Mouse infection

Mice were infected intraperitoneally with 50 CFU of *Salmonella* Typhimurium WT or *Salmonella* Typhimurium *ΔsopB*, both of which expressed green fluorescent protein (GFP). Bacteria were diluted in 100 μL of phosphate-buffered saline (PBS). Immediately after infection and during follow up, the mice were provided with 100 µg/mL ampicillin (Bristol-Myers Squibb) in their drinking water to maintain plasmid expression. At the indicated post-infection times, the mice were sacrificed and splenectomized to obtain a cell suspension for flow cytometry assays or for determining colony forming units (CFUs).

### Plasmids

The SopB-encoding gene from *Salmonella* Typhimurium 14,028 was cloned into the pcDNA6-MycHis plasmid to obtain pcDNA6-*sopB*.

### Cell infection and intracellular survival assay

B cells were infected with *Salmonella* at an MOI of 50 as have been described previously [25]. Infected cells wee resuspended in RP10 medium for 10 minutes at 37ºC and centrifuged at 1500 rpm. The supernatant was discarded, and the B cells and *Salmonella* were maintained at the bottom for an additional 20 minutes. After 30 minutes of total contact, the cells were washed twice with PBS-gentamicin (80 µg/mL) (Sigma-Aldrich, G1914) to eliminate all extracellular bacteria. After the second wash, the infected cells were suspended in RP10 medium with gentamicin (80 µg/mL). During the incubations, the cells were kept at 37ºC in a humidified incubator with 5% CO_2_. To measure intracellular bacteria at 1, 3 and 24 hours post-infection, the B cells were lysed with 1% Triton X100 (Sigma-Aldrich, X100), and the dilutions were plated onto LB agar to determine CFUs [60].

### Cytokine assays

The levels of IL-1β, IL-6 and IL-10 in culture supernatants were determined using a Mouse Cytokine Magnetic Bead Panel (Milliplex, MCYTOMAG-70K). The levels of IL-18 in the supernatants were determined using a quantitative sandwich enzyme immunoassay (MBL 7625). B cells were infected at the indicated MOI as previously described, and the culture supernatants were collected at 24 hours post-infection for the assays.

### Cytotoxicity assay

B cells were infected as previously described. Cytotoxicity was assessed twenty-four hours post-infection using a lactate dehydrogenase (LDH) release assay (Promega, G1780). The percentage of LDH release was calculated using the following formula: percentage of release = (experimental LDH release – spontaneous LDH release)/(maximal LDH release – spontaneous LDH release) x 100%.

### Western blotting analyses

B cell proteins from each experiment were obtained using an NE-PER Protein Extraction Reagent kit (Thermo Fisher, 78,833) according to the manufacturer’s instructions and were solubilized in Laemmli sample buffer. The samples were resolved in an 8% gel via SDS-PAGE and transferred to polyvinylidene difluoride (PVDF) membranes. The membranes were blocked in BSA (1%) in Tris-buffered saline (TBS) containing 0.1% Tween (TBST) and were incubated overnight at 4ºC with any of the following primary antibodies diluted in blocking buffer: anti-pAkt S473 ab (Cell Signaling, 4060S, dilution 1:1000), anti-pAkt T308 (Cell Signaling, 130,385, dilution 1:1000), anti-Akt (Santa Cruz Biotechnology, SC-8312, dilution 1:1000), anti-pYAP S127 (Cell Signaling, 4911, dilution 1:1000), anti-YAP ab (Santa Cruz Biotechnology, SC-15,407, dilution 1:1000), anti-p-p85 Y458 (Cell Signaling, 4228, dilution 1:1000), anti-p85 (Cell Signaling, 4257, dilution 1:1000) or anti-β-actin (Cell Signaling, 4970. dilution 1:3000). After primary antibody incubation and five subsequent washes with TBST, the membranes were incubated with an anti-rabbit HRP-coupled secondary antibody (Cell Signaling, 7074, dilution 1:3000). After five subsequent washes with TBST, the proteins were detected via chemiluminescence (Promega, W1001) using a ChemiDoc imaging system (BioRad Laboratories, Hercules, CA) and analyzed using ImageLab software (BioRad Laboratories).

### Immunofluorescence

B cells from each experiment were fixed for 30 minutes at room temperature using paraformaldehyde (PFA) at a final concentration of 2%. The fixed cells were washed three times with PBS to eliminate PFA, adhered to glass slides pretreated with poly-L-lysine (Sigma, P-1524) and permeabilized in methanol (10 minutes at −20ºC). After permeabilization, the cells were blocked with blocking buffer (bovine serum albumin-phosphate-buffered saline solution (PBS-BSA 1%)) for 60 minutes at room temperature. After being blocked, the cells were incubated overnight at 4ºC with any of the following antibodies diluted in blocking buffer: anti-pAkt S473 (Cell Signaling, 4060S, dilution 1:200), anti-pAkt T308 (Cell Signaling, 13038S, dilution 1:200), anti-pYAP S127 (Cell Signaling, 4911S, dilution 1:200), anti-PIP3 (Echelon, Z-P345b, dilution 1:100) or anti-Myc (Cell Signaling 9483, dilution 1:200). After primary antibody incubations and five subsequent PBS washes, the cells were incubated with the corresponding TRITC-coupled secondary antibody (anti-rabbit, Jackson ImmunoResearch, 111-025-003, dilution 1:200, anti-mouse, Jackson ImmunoResearch, 315-025-003, dilution 1:200) or FITC-coupled secondary antibody (anti-rabbit, Jackson ImmunoResearch, 711-095-152, dilution 1:200) diluted in blocking buffer for 60 minutes at room temperature. After secondary antibody incubation, the cells were washed five times with PBS and incubated in DAPI (Molecular Probes, D1306) for 20 minutes at room temperature to stain the nuclei. Images were obtained using a Plan-Apochromat 63x/1.40 Oil DIC objective in a Carl Zeiss Confocal Microscope LSM700 (Carl Zeiss Oberkochen, Germany) and were analyzed using ZEN software (Carl Zeiss) and/or ImageJ (NIH).

### Flow cytometry

Splenocytes from mice were resuspended in PBS, and Fc receptors were blocked in suspension with human γ-globulin at a final concentration of 0.02 µg/µL (Octapharma) for 20 min at 4ºC. The cells were then incubated with any of the following antibodies in 50 µL of PBS for 20 min at 4ºC: Pacific Blue-conjugated anti-CD19 (Biolegend, 115,523, dilution 1:100), anti-pAkt S473 (Cell Signaling, 4060S, dilution 1:200), anti-pAkt T308 (Cell Signaling, 13038S, dilution 1:200) or anti-pYAP S127 (Cell Signaling, 4911S, dilution 1:200). After being washed with PBS, the cells were incubated with an anti-rabbit TRITC-coupled secondary antibody (Jackson ImmunoResearch, 111-025-003, dilution 1:200) for 20 min at 4ºC. After being washed with PBS, the cells were fixed with paraformaldehyde (2%). Flow cytometry was performed on a CYAN cytometer (Beckman Coulter), and the data were analyzed using FlowJo software, version 10.0 (TreeStar).

### Transfection

Transfection of 1 × 10^6^ A20 cells (ATCC TIB-208) by electroporation was performed using the Amaxa Nucleofector 2b system (Lonza). Cells resuspended in 0.1 mL of PBS were mixed with 1.5 µg of pcDNA6-*sopB*. The cell-plasmid suspension was transferred to a new electroporation cuvette, and the cells were electroporated using the manufacturer’s program for the A20 cell line. After transfection, the cells were re-suspended in RPMI medium supplemented with FBS (10%).

### Statistical analyses

Data from at least three independent experiments were analyzed by Student’s *t*-test or two-way ANOVA with a post hoc Bonferroni test using GraphPad Prism 5.

## Acronyms and abbreviations

mTORC1: Mammalian target of rapamycin complex 1
mTORC2: Mammalian target of rapamycin complex 2
NFκB: Nuclear factor κB
NLRC4: NLR family CARD domain-containing protein 4
PDK1: Phospho-inositide dependent kinase 1
PI3K: Phosphoinositide 3-kinase
Rictor: Rapamycin-insensitive companion of mTOR
SPI: *Salmonella* pathogenicity island
STAT3: *Signal transducer and activator of transcription 3*
TRAF6: Tumor necrosis factor receptor (TNFR)-associated factor 6
T3SS: Type-three secretion system
YAP: Yes-associated protein
