# Supplementary material for: SopB activates the Akt-YAP pathway to promote Salmonella survival within B cells
Source: Virulence. 2018 Aug 26;9(1):1390–402. doi: 10.1080/21505594.2018.1509664 (PMC6177241; doi:10.1080/21505594.2018.1509664)
Supplement: Supplemental Material [file kvir-09-01-1509664-s001.pdf]

Suplementary figures

Abraham Garcia-Gil, Carlos Samuel Galan-Enriquez, Araceli Perez-Lopez, Porfirio Nava, Celia Alpuche-Aranda, Vianney Ortiz-Navarrete

A

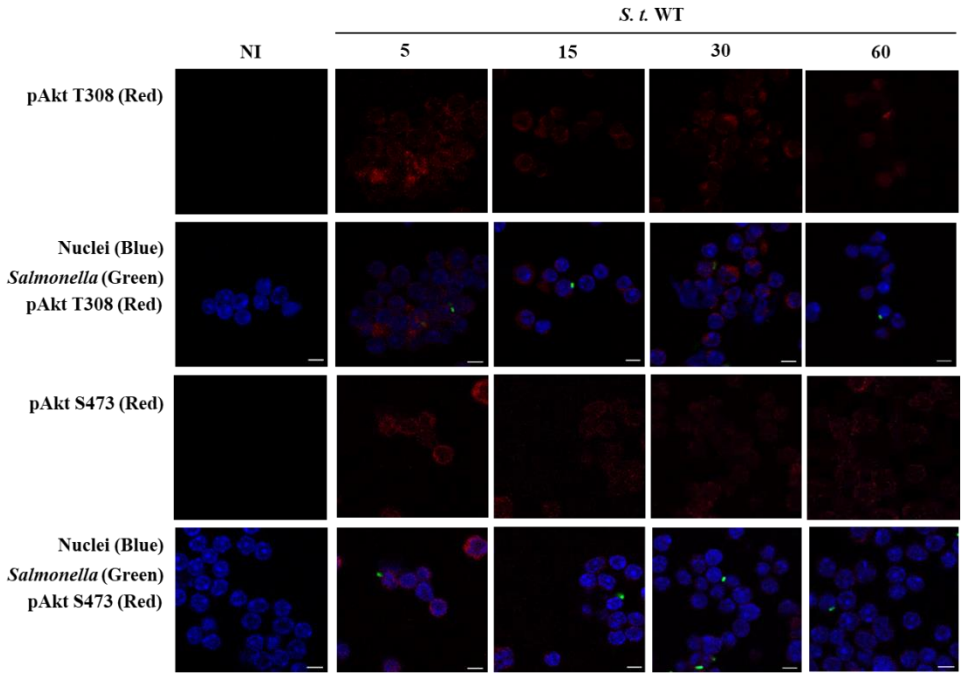
S. t. WT
NI
5
15
30
60
pAkt T308 (Red)
Nuclei (Blue)
Salmonella (Green)
pAkt T308 (Red)
pAkt S473 (Red)
Nuclei (Blue)
Salmonella (Green)
pAkt S473 (Red)

B

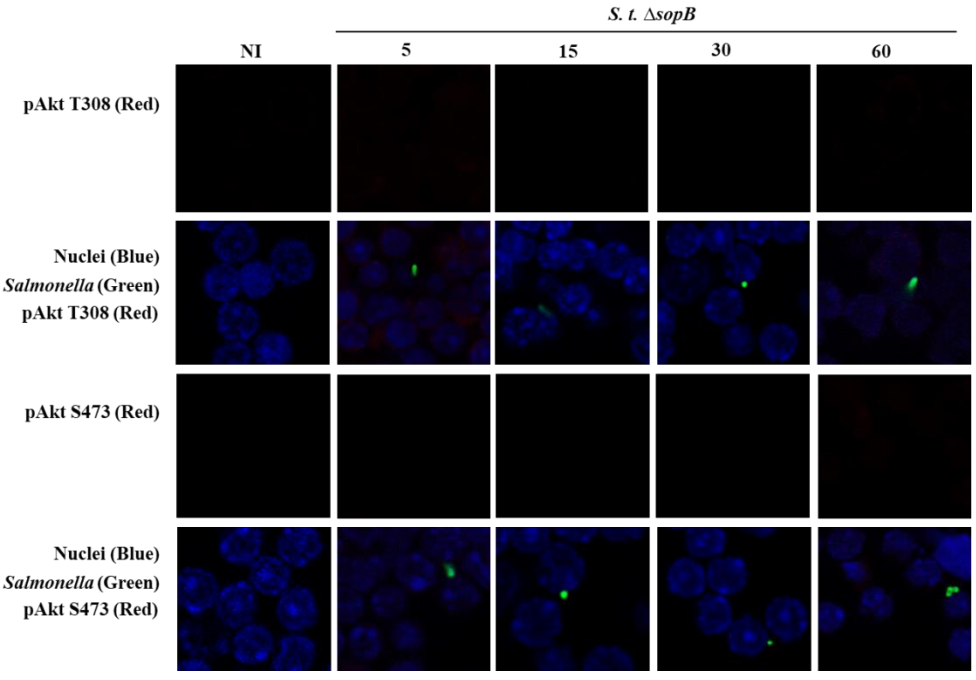
S. t. ΔsopB
NI
5
15
30
60
pAkt T308 (Red)
Nuclei (Blue)
Salmonella (Green)
pAkt T308 (Red)
pAkt S473 (Red)
Nuclei (Blue)
Salmonella (Green)
pAkt S473 (Red)

A

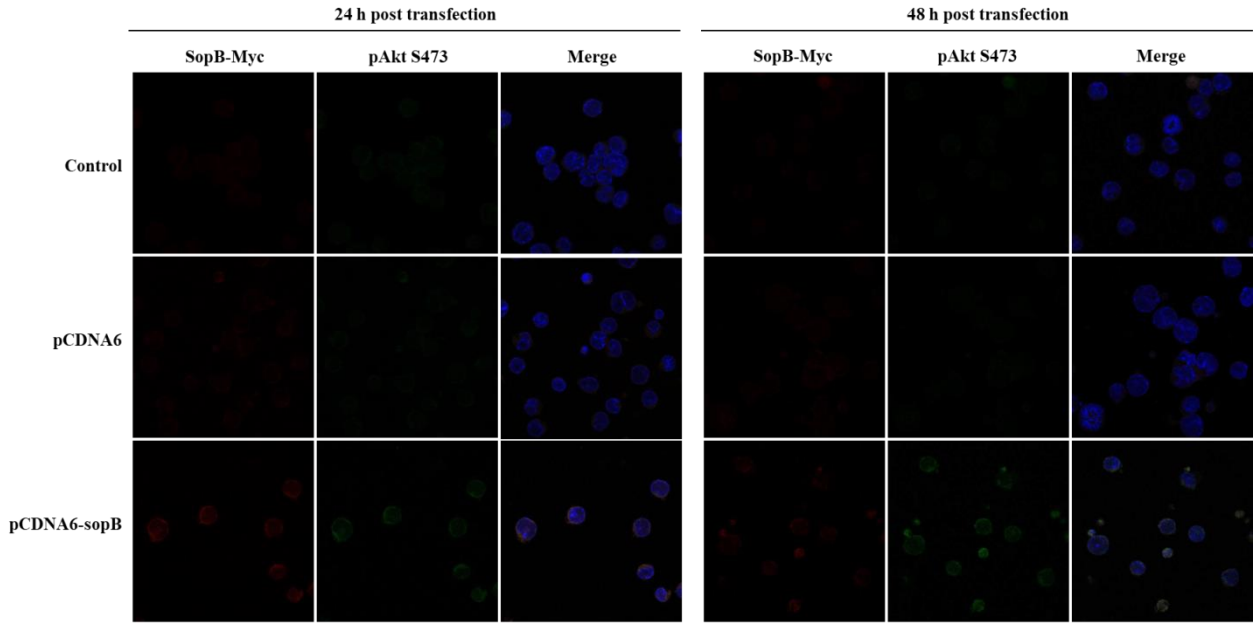
24 h post transfection
48 h post transfection
SopB-Myc
pAkt S473
Merge
SopB-Myc
pAkt S473
Merge
Control
pCDNA6
pCDNA6-sopB

B

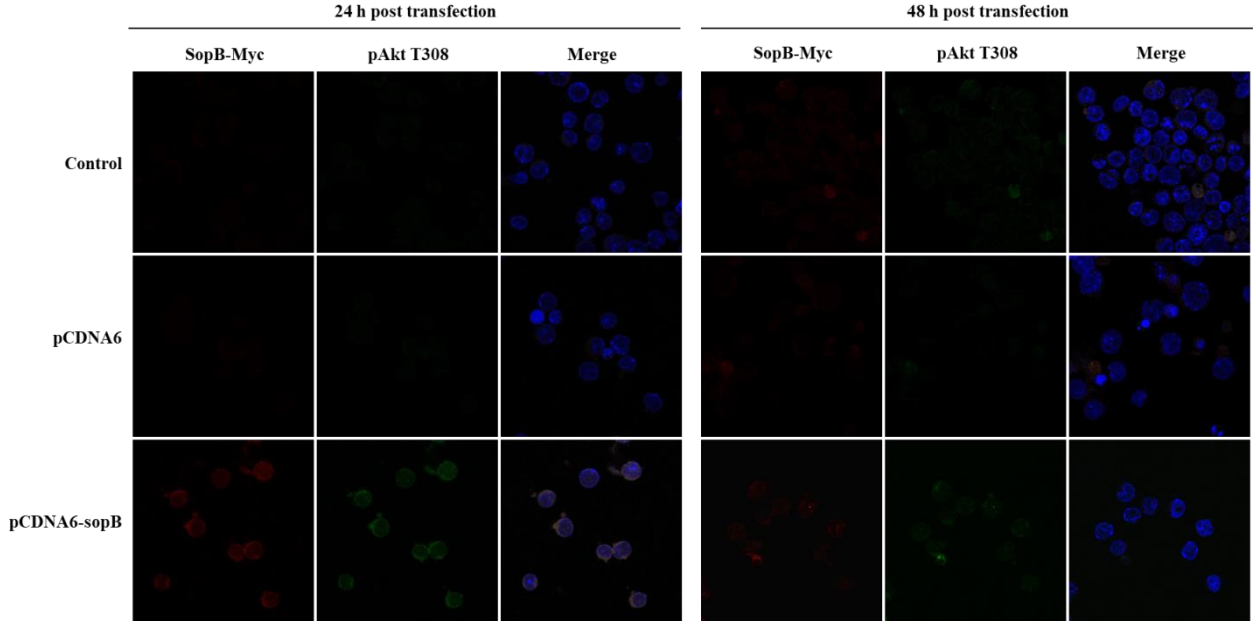
24 h post transfection
48 h post transfection
SopB-Myc
pAkt T308
Merge
SopB-Myc
pAkt T308
Merge
Control
pCDNA6
pCDNA6-sopB
